# Supplementary material for: Chromothripsis during telomere crisis is independent of NHEJ, and consistent with a replicative origin
Source: Genome Res. 2019 May;29(5):737–49. doi: 10.1101/gr.240705.118 (PMC6499312; doi:10.1101/gr.240705.118)
Supplement: Supplemental Material [file supp_gr.240705.118_Supplemental_file_1.zip › contigs/annotated_contigs/DB56/contig.2.DB56_length_544_mean_cov_5.79044117647.docx]

**DB56_length_544_mean_cov_5.79044117647**

```
AGAGCACCCAAGAGATTATGTCTTTTGACTTCAGCTACCAGGGCAAGTAGAGAAAGACCATCAAGTGCAGGCAGGGTTAGACATGTCTG
 >chr14:87036798-87037072 + E=3e-150
AGCTCAGACTCTCCTTGGGCAGGGCTTGCTTTGGCAGCTGTGGGGACGGGTGTGTGGTTCTCAGGCCAGTGAAGTTATGTTTCCAGGAG

GGGATTATGGCTGCTGTTGCTGTGTCATACAGGTTTCCAGGGAAGTAGGGAAAAGCCAGTAGTGACAGGCCTCACCCAGGTTCCATGCA

GCCAG|CA|TTTTTCTTCCCTAGGACAATACCCTGAAATGCTCGAGTTTGTGTGCCTTCTTCCAGTCCTGCAGGGTCAAGCCAGTTGTC
      >chr14:63623899-63624171 + E=5e-152
AGCCAGGACACATGTACTTGCTTTCCATGAGGGTATGCTCAGAGAACCGGACTGGAGTAGGGGGAAGAAGCATGCAGAGTGTTTGGGGG

ACTTGTGGGCCAGCTGGAGGTGGGGTACACAGGCAAGGCCTCCTGGGTGGCTGTAGTCAGGCTTCCTGAGGGAGTTTGTAGGGCAGTTA

GCAAGGTTTGCA
```
